# Changes in inflammatory parameters and their impact on clinical symptoms in patients suffering from schizophrenia

**DOI:** 10.1192/j.eurpsy.2023.329

**Published:** 2023-07-19

**Authors:** B. Nycz, K. Krysta

**Affiliations:** Department of Rehabilitation Psychiatry, Medical University of Silesia, Katowice, Poland

## Abstract

**Introduction:**

One of the factors influencing the symptoms of schizophrenia, which may indicate changes in the cognitive functioning of patients, is the fluctuating level of inflammatory cytokines.

**Objectives:**

The aim of the review was to analyze the available literature on the importance of selected inflammatory factors [interleukin-1β (IL-1β), interleukin-6 (IL-6), interleukin-8 (IL-8), interleukin-10 (IL-10), of tumor necrosis α (TNF-α)] in schizophrenia and the assessment of the impact of changes in cytokine levels on the occurrence of schizophrenia symptoms.

**Methods:**

For this purpose, available scientific publications from following databases: PubMed, Scopus, Google Scholar were used to prove that the levels of selected inflammatory parameters changed in people suffering from schizophrenia. Moreover, fluctuations in cytokine concentrations influenced the occurrence of negative symptoms of schizophrenia, including cognitive disorders, as well as psychotic symptoms.

**Results:**

An increase in the concentration of IL-1β in the cerebrospinal fluid of patients with the first episode of schizophrenia has been described, which may indicate the involvement of the cytokine in the inflammatory process involving the CNS. The increased level of IL-6 is associated with the occurrence of psychotic disorders, it is also noted in stressful conditions. IL-6 is qualified as an indicator of exacerbation of schizophrenia, which normalizes after antipsychotic treatment. In the blood of patients with paranoid schizophrenia, elevated levels of IL-8 and IL-6 were detected compared to healthy individuals, which indicates the development of an inflammatory process in schizophrenia. The relationship between the level of IL-8 in women in the second trimester of pregnancy and the risk of developing schizophrenia spectrum disorder in children has been proven. Untreated patients with acute psychotic symptoms showed an increase in the level of TNF-α in the blood serum (compared to healthy subjects). An increase in the level of TNF-α in the blood serum of patients with an acute relapse of schizophrenia or the first episode of psychosis was also demonstrated. In conclusion, the relationship of IL-6 and TNF-α with the occurrence of psychotic disorders, the relationship of IL-1β with the appearance of changes in mood, behavior, including cognitive dysfunction, the relationship of IL-8 with the risk of developing schizophrenia spectrum disorder in children, the relationship of reduced concentrations of IL-10 with the intensification of negative symptoms, including cognitive deficits.

**Conclusions:**

In conclusion, the analysis showed that patients with schizophrenia fluctuate in the concentration of inflammatory cytokines, which affects the occurrence of clinical symptoms.

**Disclosure of Interest:**

None Declared

